# The metabolomics profile of growth rate in grazing beef cattle

**DOI:** 10.1038/s41598-022-06592-y

**Published:** 2022-02-15

**Authors:** José Augusto Imaz, Sergio García, Luciano Adrián González

**Affiliations:** Sydney Institute of Agriculture, School of Life and Environmental Sciences, Faculty of Science, The University of Sydney, Sydney, NSW 2570 Australia

**Keywords:** Animal physiology, Metabolomics

## Abstract

This study aimed to determine the relationship between the metabolome and changes in growth rate (i.e., liveweight change, LWC) and molasses-lick block supplement intake (MLB) of grazing cattle. Weaner beef cattle were fed for 220 days with a sequence of feed types and blood samples, growth rate, and supplement intake were taken on five points in time. The relative abundance (RA) of plasma metabolites were determined using proton nuclear magnetic resonance (NMR). Sixty-four per cent of the metabolites identified were associated with LWC but only 26% with MLB intake (*P* < 0.05). Periods with faster growth rate showed high availability of amino acids (i.e., valine, leucine, isoleucine, phenylalanine and tyrosine), acetate, and 3-hydroxybutyrate. Periods with lower growth rate were associated with high RA of lipids, choline and acetate. The metabolic profile of individual animals during a period of compensatory growth (after periods of poor performance) showed that high-performing animals were characterised by lower RA of amino acids (i.e., valine, leucine, isoleucine, methylhistidine), creatinine, creatine, pyruvate, 3-hydroxybutyrate, and acetyl groups. It is speculated that high-performing animals have faster uptake of these metabolites from the bloodstream. Cattle growth rate over time was associated with their metabolome which could be used to ensure that the availability of certain metabolites promoting growth is tailored in feed supplements to improve production.

## Introduction

Live weight change (LWC; i.e., growth rate) could be considered one of the most critical descriptors of performance in grazing beef cattle^1–3^ and has proved to be affected by nutrition^4^, diseases^5^ and welfare^6^. However, the metabolic pathways behind growth responses are far from being fully understood^7^. This is particularly evident in grazing beef cattle as producers feed them with different types of forages varying in quantity and quality over seasons^8^ and a large variation in growth rate amongst individual animals exist^9^. As a result, associations between changes in the metabolism of grazing animals and their LWC over time and amongst individuals at different points in time were poorly explored^7,10^.

Nowadays, in-paddock technologies enable real-time monitoring of grazing cattle performance, remotely and automatically^11,12^. For instance, automatic weighing scales (WOW) can be used to measure live weight (LW) and electronic feeders (EF) to measure supplement intake, even of those consumed in small quantities such as molasses-lick blocks (MLB)^9^. Therefore, the use of these technologies could enable a simultaneous assessment of the relationship between the blood metabolome and LWC and supplement intake in grazing cattle fed as a group. Previous studies in beef cattle reported that animals’ metabolic profile is linked to feed efficiency^13^, carcass quality^14^ and LW^15^. Nevertheless, these studies assessed cattle metabolome at a single point in time whereas animals’ performance was measured or averaged over long periods^14,15^. Therefore, the relationship between the metabolome of grazing beef cattle with LWC and supplement intake has not been studied at similar time scale to the authors’ best knowledge. Interestingly, metabolic pathways affecting LWC could highlight metabolites of low molecular weight (e.g., sugars, lipids, amino acids) involved in complex interactions between external (e.g., nutrition) and internal (e.g., genotype) factors expressing the phenotype^7,16^. This knowledge can be used to enhance and tailor the nutritional management of animals^17^ and biomarker discovery of desirable economic traits^13^.

The present study aimed to investigate the associations between the relative abundance (RA) of blood metabolites in grazing beef cattle and their LWC and MLB intake as animals grazed different forages over time. We hypothesised that LWC and the intake of MLB are reflected in significant changes in the blood metabolome of animals.

## Results

### Live weight change, live weight, and molasses-lick block intake

**Figure 1:**
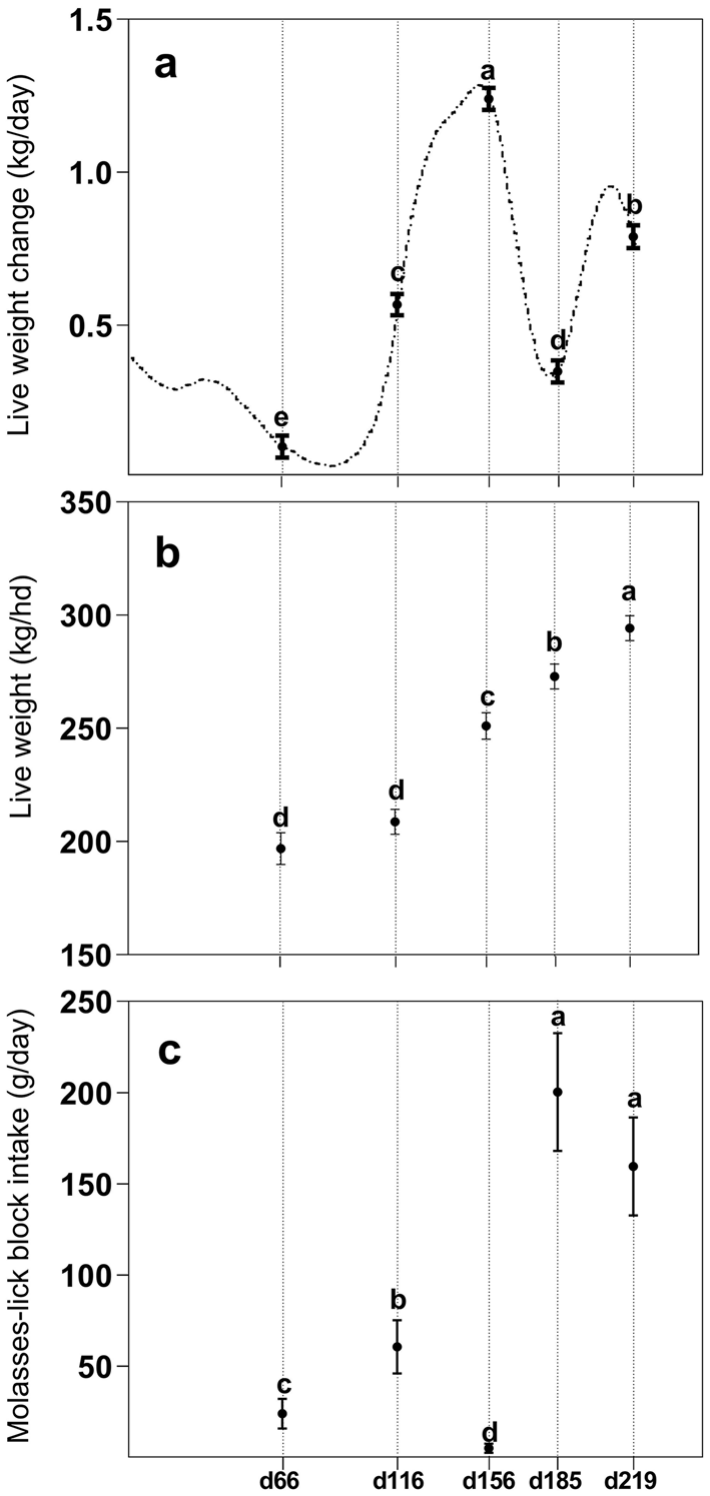
(**a**) Daily live weight change (**b**) Live weight measured with an automatic weighing system and (**c**) molasses-lick block intake of grazing weaner cattle over time. Continuous line in panel (**a**) indicates the average live weight change (LWC) of the herd.

Average LWC was different between sampling dates with the greatest LWC occurring at d156 and the lowest at d66 while animals were grazing pastures (Fig. 1, panel a; *P* < 0.05). A large LWC variability between individual animals was observed over time, with values ranging from − 0.55 to 1.80 kg/d. In addition, average LW varied from 196 ± 7.1 to 294 ± 5.5 kg/hd at d66 and d219, respectively (Fig. 1, panel b, *P* < 0.05). Molasses-lick block intake was affected by Day being highest at Day 185 and 219 (Fig. 1, panel c; *P* < 0.05). The intake of MLB also showed a large variability from 0 to 780 g/d amongst animals and sampling dates (Data not shown; *P* < 0.05). Live weight change and MLB intake were not affected by Sex (*P* > 0.05).

### Principal component analysis with the relative abundance of metabolites

The RA of all metabolites was affected by sampling day (Table 1, *P* < 0.05). Interestingly, the RA of several amino acids such as valine, leucine, isoleucine and tyrosine, and dimethyl sulfone and 2-hydroxybutyrate, was highest at d156 when LWC was also at its peak (Fig. 1, panel a). Conversely, creatinine showed the lowest RA at d156 whereas acetate peaked at d219 when oaten hay was the main feedstuff (*P* < 0.05). The RA of 3 hydroxybutyrate, glucose, glutamine, and glycine and acetyl groups was highest at d116. In contrast, the RA of very-low-density lipids (VLDL), low-density lipids (LDL) and unsaturated was lowest at d116 (*P* < 0.05). There were significant interactions Day x Sex (*P* < 0.05) for glucose and acetate with greater RA for steers at d156 and d219, respectively, and the RA of choline was greater in heifers at d156 (Data not shown).

**Table 1:** Relative abundance of blood metabolites on five sampling points of grazing beef cattle and the *P*-values for factors in the statistical model blood sampling day (Day), live weight change (LWC) as a covariate, and their interactions.

Metabolite	d66	d116	d156	d185	d219	Day	LWC	Day x LWC
Valine	504.2 ± 6.83 c	603.5 ± 6.83 b	672.1 ± 6.83 a	593.5 ± 6.83 b	594.5 ± 6.83 b	< 0.01	< 0.01	< 0.001
Leucine	124.4 ± 1.96 d	129.4 ± 1.92 cd	165.4 ± 1.92 a	147.6 ± 1.92 b	136.8 ± 1.92 c	< 0.01	0.122	< 0.001
Acetate	285.1 ± 10.23 b	181.3 ± 10.12 c	282.5 ± 10.12 b	201.5 ± 10.12 c	412.5 ± 10.12 a	< 0.001	0.54	< 0.001
Very-low density lipids	276.1 ± 4.54 a	157.7 ± 4.54 d	221.2 ± 4.54 c	260.9 ± 4.54 ab	247.7 ± 4.54 b	< 0.001	0.034	< 0.001
Unsaturated lipid	166.6 ± 3.66 a	81.0 ± 3.66 c	137.8 ± 3.66 b	169.8 ± 3.66 a	131.3 ± 3.66 b	< 0.001	< 0.001	< 0.001
Pyruvate	53.6 ± 0.99 b	61.9 ± 0.99 a	52.9 ± 0.99 b	50.1 ± 0.99 c	52.3 ± 0.99 b	< 0.001	0.791	< 0.01
Acetyl groups	219.5 ± 3.23 b	257.2 ± 3.23 a	222.2 ± 3.22 b	199.8 ± 3.22 c	226.2 ± 3.22 b	< 0.001	0.046	< 0.01
Creatinine	41.3 ± 0.68 a	38.5 ± 0.68 b	32.3 ± 0.68 c	37.8 ± 0.68 b	41.7 ± 0.68 a	< 0.001	< 0.001	< 0.01
Alanine	94.6 ± 1.17 ab	95.3 ± 1.15 ab	97.1 ± 1.15 a	93.3 ± 1.15 ab	91.3 ± 1.15 b	< 0.001	0.101	< 0.01
Choline	93.6 ± 1.22 a	72.2 ± 1.22 d	81.7 ± 1.22 c	87.1 ± 1.22 b	90.4 ± 1.22 ab	< 0.001	0.196	< 0.01
Methylhistidine	26.4 ± 0.50 b	25.5 ± 0.50 b	29.6 ± 0.50 a	28.9 ± 0.48 a	29.7 ± 0.48 a	< 0.01	< 0.01	< 0.01
2-hydroxybutyrate	135.3 ± 1.91 c	156.4 ± 1.87 b	173.3 ± 1.89 a	160.1 ± 1.87 b	157.2 ± 1.87 b	< 0.001	< 0.01	0.011
Citrate	134.1 ± 3.57 d	170.35 ± 3.54 bc	165.6 ± 3.54 c	185.1 ± 3.54 a	176.2 ± 3.54 ab	< 0.001	0.09	0.012
Low-density lipids	143.2 ± 1.83 ab	117.0 ± 1.85 c	137.3 ± 1.81 b	143.0 ± 1.81 ab	147.2 ± 4.41 a	< 0.001	0.221	0.013
3-hydroxybutyrate	300.1 ± 6.06 c	348.8 ± 6.06 a	335.4 ± 6.06 a	312.0 ± 6.06 c	332.4 ± 6.06 a	< 0.001	0.059	0.017
Creatine	89.8 ± 2.48 c	116.5 ± 2.51 b	119.5 ± 2.48 ab	121.9 ± 2.48 ab	126.4 ± 2.40 a	< 0.001	< 0.01	0.021
Isoleucine	73.1 ± 1.36 d	83.0 ± 1.38 c	109.3 ± 1.36 a	90.7 ± 1.36 b	84.7 ± 1.36 c	< 0.001	0.562	0.07
Phenylalanine	37.4 ± 0.64 b	38.9 ± 0.64 b	43.8 ± 0.63 a	37.8 ± 0.63 b	42.2 ± 0.63 a	< 0.001	0.11	0.124
Formate	9.1 ± 0.29 b	10.6 ± 0.28 a	8.4 ± 0.28 b	5.9 ± 0.28 c	10.7 ± 0.28 a	< 0.001	0.306	0.244
Lactate	111.6 ± 4.40 a	111.3 ± 4.40 a	83.9 ± 4.40 b	91.3 ± 4.40 b	87.9 ± 4.40 b	< 0.001	0.481	0.295
Glutamine	228.8 ± 3.24 bc	272.6 ± 3.24 a	238. 3 ± 3.24 b	217.8 ± 3.24 c	238.8 ± 3.24 b	< 0.001	0.681	0.325
Glucose	2823.0 ± 38.40 e	3976.1 ± 38.40 a	3471.1 ± 37.40 b	3213.3 ± 37.40 c	2997.6 ± 37.40 d	< 0.001	0.19	0.416
Mannose	12.7 ± 0.24 c	15.9 ± 0.25 a	14.5 ± 0.24 b	15.6 ± 0.24 a	12.5 ± 0.24 c	< 0.001	< 0.01	0.455
Tyrosine	29.7 ± 0.59 bc	28.2 ± 0.59 cd	37.1 ± 0.59 a	27.1 ± 0.59 d	31.0 ± 0.58 b	< 0.001	0.629	0.548
Threonine	71.3 ± 1.47 c	82.3 ± 1.47 a	74.9 ± 1.47 bc	74.4 ± 1.47 bc	78.3 ± 1.47 ab	< 0.001	0.931	0.65
Dimethyl sulfone	27.9 ± 2.23 d	37.6 ± 2.21 c	110.2 ± 2.21 a	41.6 ± 2.21 bc	46.5 ± 2.21 b	< 0.001	0.037	0.79
Glycine	125.0 ± 3.84 d	223.1 ± 3.79 a	152.3 ± 3.80 c	179.6 ± 3.80 b	152.2 ± 3.72 c	< 0.001	< 0.01	0.79

^a,b,c,d,e^ Means within rows without a common superscript differ.

**Figure 2:**
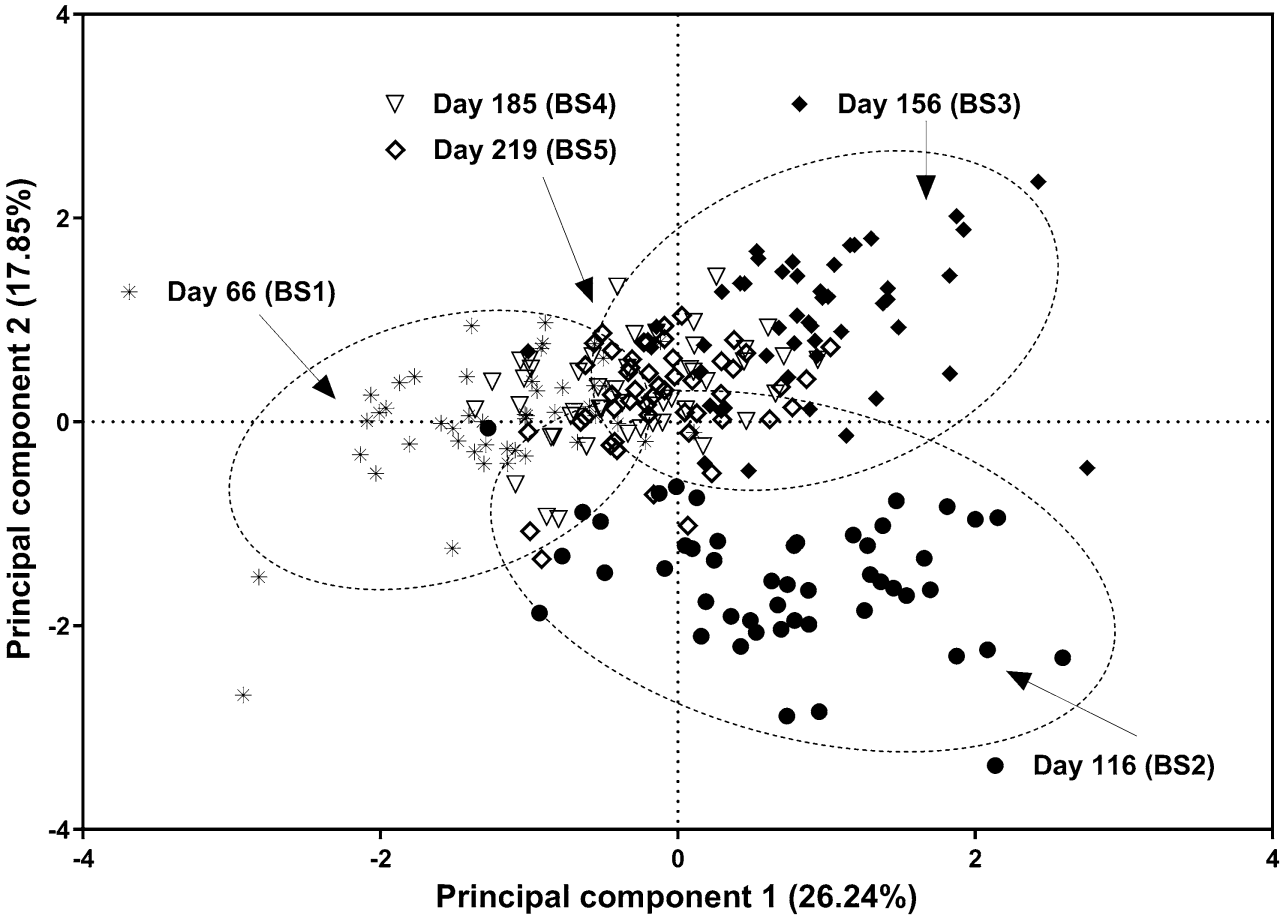
Score plot from principal component analysis of 27 blood metabolites of grazing beef cattle.

The score plot from PCA presented in Fig. 2 shows that data points clustered together according to sampling day with the first two PC explaining 44% of the variability. Samples from d116 clustered in the lower right quadrant of the plot due to positive values for PC1 and negative values for PC2. Samples from d66 (lowest LWC) showed negative values for PC1, but PC2 were negative and positive. In contrast, samples on d156 (highest LWC) clustered together with positive values for both PC1 and PC2. Principal component scores for samples on d185 and d219 clustered in the centre of the quadrants around zero.

**Figure 3:**
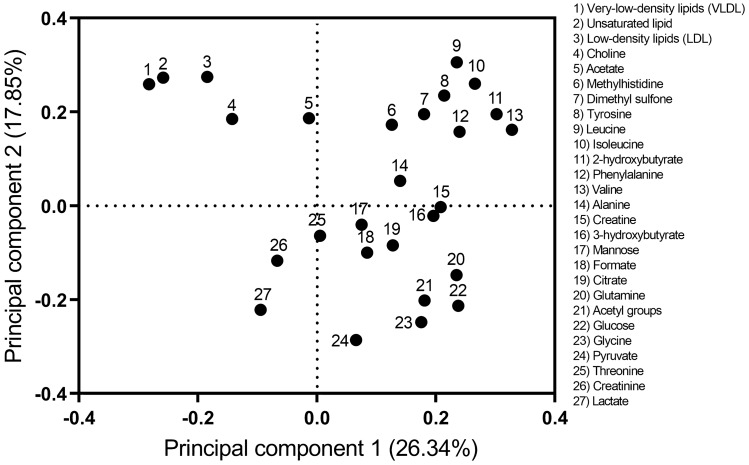
Loading plot from principal component analysis of 27 blood metabolites of grazing beef cattle.

The loading plot in Fig. 3 showed that groups of metabolites clustered in different regions of the plot. Lipid groups and choline clustered in the quadrant with negative PC1 and positive PC2. The quadrant with positive values for both PC1 and PC2 was represented by dimethyl sulfone, 2-hydroxybutyrate, and a group of amino acids (methylhistidine, tyrosine, leucine, isoleucine, phenylalanine and valine). Glutamine, acetyl groups, glucose, glycine and pyruvate showed positive loading on PC1 but negative on PC2.

### Associations between the metabolome and performance

The RA of 16 out of 27 metabolites identified were affected by the Day x LWC interaction (Table 1; *P* < 0.05). Also, dimethyl sulfone was positively associated with LWC independently of sampling time (Table 1, *P* < 0.05; β = 6.98 ± 3.38; *P*-value 0.037) whereas glycine (β =  − 19.05 ± 5.510; *P* < 0.01) and mannose (β =  − 1.40 ± 0.328; *P* < 0.01) were negatively associated with LWC (Table 1, *P* < 0.01).

Significant regression coefficients (covariates) were found on d116 (56%) when LWC was in an upwards trajectory after a period with low or nil LWC (Table 2; *P* < 0.05). Most of these significant Day x LWC interactions at d116 were due to a negative association between the RA of metabolites and LWC, except for choline and lipids that showed a positive association (Table 2; *P* < 0.05). Creatinine showed negative associations with LWC at d66, d116, d185 and d219 whereas creatine was negatively associated with LWC on d116 and d185 (*P* < 0.05). Valine, leucine, isoleucine, 2- and 3-hydroxybutyrate, citrate and pyruvate showed a negative regression coefficient on d116; however, acetate showed a positive association with LWC at d219 (*P* < 0.05).

**Table 2:** Regression coefficient (β), intercept (α) and *P*-value for the intercept and regression coefficient between the relative abundance of blood metabolites and live weight change (LWC) on each of five points (Day). Only metabolites with a Day x LWC interaction are shown (*P* < 0.05).

Blood sampling day (Day)	Metabolite	Intercept			Regression coefficient		
		α	SE	*P*-value	Β	SE	*P*-value
d66	Creatinine	42.68	0.819	< 0.001	− 11.65	4.265	< 0.01
d116	Very low− density lipids	118.29	9.359	< 0.001	82.22	17.084	< 0.001
	Unsaturated lipid	45.43	6.943	< 0.001	70.57	12.629	< 0.001
	Low-density lipid	105.88	4.060	< 0.001	22.75	7.371	< 0.01
	Choline	61.84	2.697	< 0.001	20.21	4.894	< 0.001
	Methylhistidine	31.27	1.137	< 0.001	− 9.95	2.103	< 0.001
	Creatinine	43.67	1.305	< 0.001	− 10.76	2.375	< 0.001
	Isoleucine	88.86	2.929	< 0.001	− 11.30	5.434	0.038
	Pyruvate	67.50	2.094	< 0.001	− 11.47	3.686	< 0.01
	Leucine	144.35	4.053	< 0.001	− 31.39	7.507	< 0.001
	Acetyl groups	273.29	5.792	< 0.001	− 31.99	10.024	< 0.01
	2-hydroxybutyrate	168.97	3.929	< 0.001	− 32.60	7.289	< 0.001
	Citrate	195.11	7.580	< 0.001	− 43.51	13.822	< 0.001
	Creatine	139.51	5.299	< 0.001	− 49.78	9.797	< 0.01
	3-hydroxybutyrate	392.71	12.515	< 0.001	− 94.51	23.565	< 0.001
	Valine	662.70	14.711	< 0.001	− 117.00	27.275	< 0.001
d156	Pyruvate	42.27	3.954	< 0.001	8.57	2.975	< 0.01
d185	Unsaturated lipid	159.36	4.841	< 0.001	32.63	10.754	< 0.01
	Creatinine	39.51	0.893	< 0.001	− 4.90	1.799	< 0.01
	Creatine	128.25	3.492	< 0.001	− 18.45	7.625	0.025
d219	Acetate	313.52	23.554	< 0.001	107.10	24.816	< .0001
	Alanine	79.75	2.588	< 0.001	13.53	2.718	< .0001
	Creatinine	45.14	1.326	< 0.001	− 3.92	1.390	< 0.01
	Acetyl groups	255.05	5.853	< 0.001	− 33.72	5.947	< .0001

The intake of MLB did not affect averaged LW and LWC (*P* > 0.10). The RA of lipid groups were affected by MLB intake regardless of Day (*P* < 0.05; Data not shown) with a positive association between MLB intake and the RA of VLDL (β = 41.73 ± 18.00; *P* = 0.021) and unsaturated lipids (β = 34.52 ± 13.41; *P* = 0.011). Additionally, dimethyl sulfone, creatine and lactate showed a Day x MLB intake interaction with negative regression coefficients between MLB intake and the RA of creatine at d66 (β =  − 211.3 ± 96.75; *P* = 0.031) and d116 (β =  − 117.4 ± 30.56; *P* < 0.01), lactate at d66 (β =  − 795.5 ± 352.16; *P*-value 0.025) and dimethyl sulfone at d219 (β =  − 29.8 ± 13.21; *P* value < 0.01). In contrast, acetate was positively correlated with MLB intake at d219 (β = 320.9 ± 61.50; *P*-value < 0.001).

## Discussion

The present study aimed to investigate the associations between the RA of blood metabolites in growing beef cattle and changes in LWC and the intake of MLB. We hypothesised that the variability in LWC and MLB intake is associated with changes in the metabolome. Far from being a fully controlled metabolic study of housed cattle, the novelty of the present study lies in detecting complex metabolic interactions occurring in grazing cattle. With this aim, the use of in-paddock technologies in the present study was critical to monitor cattle LWC and MLB intake enabling the integration of the three data streams at a given point in time for individual animals grazing as a group. Previous studies have indicated that associations between the RA of blood metabolites and LWC were affected by animal traits (breed, LW, age, beef, or dairy production), feeding management (grazing pastures, feed lotting), genetics and experimental design^14,15,18–22^. However, the present study is the first to assess the metabolome and performance of grazing beef cattle simultaneously.

There were higher number and stronger associations between the RA of metabolites and LWC compared to those observed with MLB intake. Thus, 64% of the metabolites were associated with LWC but only 26% with MLB intake. A potential explanation for this could be that MLB intake was generally low and the changes in the metabolome may have been overridden by LWC. However, these results demonstrated that even a small intake of self-fed supplements was reflected in the blood metabolome which may be useful to understand animals’ response to feed supplementation. Nevertheless, results from the present study should be interpreted with caution because individual forage intake (quality and total) was not measured to assess its effects on the metabolome, which is a common limitation in ruminants kept extensively. Therefore, our outcomes should be circumscribed to the present experimental design, being results linked to a particular point in time characterised by a specific LW and LWC. However, our approach allowed individual measurements of MLB and LWC of grazing animals although further research is encouraged to account for other sources of variability in a similar time frame.

The score plot from the PCA reflected changes in LWC that were particularly evident in d66, d116 and d156 which showed contrasting and increasing LWC. Animals were going through a period of decreasing LWC at d66 with 18% of animals losing weight at this time. In contrast, animals were increasing growth rate at d116 after a period of low growth rate during the winter which is often considered compensatory growth although an exact definition or threshold of LWC for compensatory growth seems lacking in the scientific literature^23^. At d156, animals were at the highest growth rate commonly observed during the spring. Interestingly, the RA of amino acids (i.e., valine, leucine, isoleucine, phenylalanine, tyrosine and methylhistidine) and dimethyl sulfone increased with LWC peaking at d156. These associations were also evident in both score and loading plots where data points from d156 and these metabolites clustered together in the upper-right quadrant. These findings demonstrate that the extent of LWC is linked with the RA of these metabolites in the bloodstream. Branched amino acids such valine, leucine and isoleucine participate in metabolic pathways related to muscle growth, protein synthesis, lipogenesis and lipolysis in animals and humans^24^. Thus, the availability of these amino acids in the diet could subsequently limit or increase its absorption and affect LWC^25^. Furthermore, it has been reported that valine and leucine increased growth rate in pigs and beef cattle^26,27^. Additionally, dimethyl sulphone has been linked to sulfur amino acids, such as methionine, which are hydrolysed to dimethyl sulphide and then oxidised to dimethyl sulfone^28^. Its abundance in ruminal fluid was positively correlated with crude protein in diet^20^. Therefore, the increased RA of dimethyl sulfone during periods of fast growth rate in the present study may reflect greater absorption of sulfur amino acids during those periods. A complex relationship exists between metabolic processes such as the absorption of metabolites from the gastrointestinal tract, its uptake and production by different tissues and organs. However, further research is needed to segregate and understand potential mechanisms driving changes in metabolites’ abundance. For example, controlled experiments could aim to manipulate growth rate by delivering the same feed quantity to individuals while altering the feed quality. Also, different animal genetics could be tested delivering the same feed quantity and quality. The integration of this knowledge could lead to tailoring feed supplements and furnish metabolites that are limiting and according to animal breed, feed being offered and nutritional requirements by cattle. For example, if a particular amino acid is limiting growth then this could be added to the feed supplement at higher concentrations.

The lowest LWC was observed on d66 where the PCA showed negative scores for PC1 driven by high RA of lipid groups (VLDL, LDL and unsaturated), choline and acetate. In addition, the RA of these lipids and choline was the highest on d66. Such high circulating concentrations of lipids may be due to mobilisation of body fat reserves during periods of low energy balance as previously reported in cattle^29,30^. In addition to the increase in the RA of choline and lipids on d66, the RA of both metabolite groups were positively associated with LWC on d116 when animals were in compensatory growth suggesting lipid metabolism is different in higher-performing animals during such periods. Interestingly, choline did cluster with lipids in the upper-left quadrant of the loading plot. Mechanisms by which choline improves cattle growth are not fully understood ^31^ but are possibly related to its role in lipid mobilisation, transport, or both^32^. The present study did not aim to investigate interactions between the RA of choline and lipids, however, our results suggest an association between them impacting cattle growth rate in agreement with other studies^31,33^. Additionally, the use of acetate in energy metabolism under contrasting growth stages may be evident at d66 and when exploring associations at each point in time as animals with faster LWC at d219 had higher RA of acetate in the bloodstream^34^. In this sense, animals experiencing low energy balance may use acetate to form Acetyl CoA by coenzyme-A, which then enters the tricarboxylic acid cycle (TAC) to produce energy via lipid oxidation although acetate can also be used to synthesise fat when the energy balance is positive^29,35^. Both of these mechanisms likely had a role in the results of the present study but these are speculations and further studies are required to elucidate the metabolic pathways of acetate depending on energy balance, current LWC and previous growth trajectory in grazing cattle.

Animals resumed growth at d116 with the score plot showing positive values for PC1 and negative for PC2 and the loading plot suggests that metabolites of influence in these results were those involved in carbohydrate metabolism (i.e. glucose, pyruvate, 3-hydroxybutyrate and acetyl groups), glutamine and glycine^23^. These metabolites also showed the highest RA on d116. The role of glucose and pyruvate as energy sources in ruminants is well known, particularly during compensatory growth^23^. However, the present study also revealed that 3-hydroxybutyrate may play an important role as a versatile metabolite contributing to the energy budget in grazing cattle. Under positive energy balance, 3-hydroxybutyrate can be esterified to fatty acids, triacylglycerides and phospholipids used for fat deposition^36^. However, 3-hydroxybutyrate could also be produced from lipolysis under negative energy balance and enter the TCA to form acetyl-CoA and produce energy^22^, which also depends on the availability of acetyl groups. Additionally, the RA of creatinine was negatively associated with LWC at d116. Creatine and creatinine are involved in energy metabolism in muscles and the brain where the intermediary form is creatine phosphate serving as an energy store in a reversible reaction that can recycle energy^37,38^. It is speculated that the ability of individual animals to produce and recycle energy via this metabolic pathway partly explains the greater performance of some animals compared to others within the same group during compensatory growth as found in the present study. Our findings could contribute to a better understanding of compensatory growth often experienced by grazing cattle due to seasonal changes in feed quantity and quality^39^.

Molasses-lick block supplementation offered in the present study provided energy from molasses, vegetable oil and by-pass protein from cottonseed meal. However, the main reason for its inclusion in grazing systems is to stimulate total intake of forage rather than contributing with large amounts of energy or protein ^9^, which is reflected by the low MLB intake of the present study. Molasses-lick-block intake variability amongst animals from the present study and the impacts of forage quality and quantity on its intake under different feed types were discussed by ^9^ and ^39^, respectively. This could partly explain the low number of associations between the RA of metabolites and MLB intake observed in the present study. Positive associations between VLDL, unsaturated lipids and MLB intake were found and it is plausible that these reflect greater absorption of lipids from the vegetable oils provided by MLB. In addition, acetate was positively associated with MLB intake at d219 when oaten hay was fed and MLB intake was high. These results could be due to MLB enhancing ruminal fermentation of fibre because of its content of by-pass protein, urea and Lasalocid as observed by ^40^ and ^41^.

The associations between the RA of metabolites and LWC within each point in time of the present study seemed to have reflected differences in the metabolic profile of individual animals. Most of the significant associations were observed at d116 with amino acids (i.e., valine, isoleucine and leucine), 3-hydroxybutyrate, pyruvate and acetyl groups being negatively correlated with LWC. We speculate that individuals with faster growth rate utilise these metabolites at a faster rate, clearing them from the bloodstream and thus showing lower RA while resuming growth at d116. However, the current experimental design does not enable to conclude on such findings because a control, well-fed group was not present at the same time. Using similar reasoning, animals with faster growth rate on d116 seemed to be able to uptake precursors of larger molecules and synthesise lipids at a faster rate compared to low-performing animals, which showed a greater abundance of lipids in their bloodstream. Connolly, et al. ^14^ found a negative association between LWC and 3-hydroxybutyrate that is in agreement with the present study but no associations with branched amino acids were observed. Nevertheless, the latter study involved feedlot cattle fed high-grain diets promoting high growth rates with fewer nutritional deficiencies in comparison with the present study using grazing animals, which usually experience periods of low feed quantity and quality. Thus, metabolites’ associations with LWC could differ in grazing cattle in comparison with cattle subjected to controlled feeding systems. However, direct comparisons of the results from the present study with previous research are difficult to establish. For instance, Connolly, et al. ^14^ averaged growth rates over 450 days whereas the present study measured growth rate in a short 3-day period, simultaneously with metabolome assessments. Therefore, findings from the present study highlight two contrasting metabolic mechanisms. One mechanism demonstrated that the metabolic profile of high performing individuals is characterised by faster uptake of metabolites of low molecular weight such as acetyl groups and 3-hydroxybutyrate which show lower RA in blood but these may be used to increase the synthesis of large molecules such as lipids which show higher RA in faster-growing animals. The other mechanism is observed across time where periods of high growth rate are characterised by high RA of metabolites with low molecular weight such as acetyl groups, 3-hydroxybutyrate, and amino acids but a low concentration of metabolites with high molecular weight such as lipids. Nevertheless, further research is needed to confirm these hypotheses along with developing novel methodologies to comprehensively account for different sources of variation in extensively kept cattle.

## Conclusion

Findings from the present study contribute to unravel the metabolism driving growth rate in grazing cattle. Our results showed that periods of high growth rate are characterised by high availability of amino acids, dimethyl sulfone, glucose, acetyl groups and 2- and 3-hydroxybutyrate whereas periods of low growth rate are characterised by high abundance of lipids, choline and acetate. Also, the metabolic profile of faster-growing animals while resuming growth rate seems to be characterised by faster uptake of amino acids (i.e., valine, leucine, isoleucine, methylhistidine), 3-hydroxybutyrate, and acetyl groups, and faster energy production and recycling via creatinine, creatine, and pyruvate. This knowledge could be utilised to improve the formulation of feed supplements to maximise growth rate.

## Methods

Fifty-two crossbred weaner cattle (Charolais × Angus; 22 heifers and 30 steers) with an initial age of 219 ± 50 days and initial body weight of 186 ± 35.1 kg/hd were fed with a range of forages for 220 days (April to November) at John Pye Farm (The University of Sydney, NSW). A two-section yard centrally located to the paddocks (15 m × 25 m) was built enclosing the only water point which contained an in-paddock WOW station with an auto drafter gate at the entry of the yard (Precision Pastoral Ltd, Alice Spring, Northern Territory, Australia). Animals were randomly assigned to one of two treatments of supplementation with molasses-lick blocks (MLB) or control but all animals grazed as one group. Treatments were applied to draft each animal upon entry to the yard either to one section containing no supplement or another section containing an electronic feeder (EF) with a single MLB offered as free-choice (Smartfeed developed by C-lock Inc., Rapid City, South Dakota, United States of America). Further details about technologies’ setup can be found in ^39^. The reporting in the present manuscript follows the recommendations of ARRIVE guidelines.

### Feeding management

Feed types consumed during the study were: (a) Autumn temperate pastures grazed from day 0 to 87 (Pastures, 7.37% CP); (b) Oat crops grazed from day 88 to 122 (OC; 10.83% CP); (c) Winter pastures with concentrate supplementation grazed from day 123 to 149 (P + C, 11.42% CP); (d) Lucerne hay fed from day 150 to 184 (LH, 21.80% CP); (e) Oaten hay fed from day 185 to 220 (OH, 7.38% CP). From day 150 to the end of the trial, animals were kept on the same paddock and fed with LH or OH because of lack of pasture growth due to drought. Additional information regarding nutritional management and chemical analysis of feed samples can be found in^9^.

### Blood sampling days (day)

Five blood samples were obtained from each animal via puncture of the coccygeal vein between 09:00 AM and 11:00 AM using evacuated tubes (containing EDTA) on days 66, 116, 156, 185 and 219 from the start of the trial (d66, d116, d156, d185, and d219, respectively). Blood samples were immediately refrigerated at 4 °C for approximately 20 min, then centrifuged (2000 × g for 30 min) and plasma was harvested and stored at − 80 °C until metabolomic analysis.

### Sample preparation for metabolome profiling

The RA of metabolites was determined by proton nuclear magnetic resonance (1H-NMR). Sample preparation and analysis were performed following published methodology by ^42^ at the facilities of Sydney Analytical (The University of Sydney, Australia).

Data were analysed using Matlab 7.0 Software (Matworks, Natick, MA). The spectra were aligned and normalised, automatically phased, baseline corrected and referenced to the α-C1H-Glucose doublet (5.233 ppm). The residual water (2.42–3.14 ppm) was truncated from the dataset to reduce analytical variability. The normalised spectra were then subjected to Standard Recoupling of Variables to obtain clusters (components or features).

The cluster value for each sample is the area under the curve for each cluster (component or peak). These values are used as relative concentrations and were multiplied by 10^6^ to reduce the number of decimal places before analysis. Also, Chenomx®, existing literature, and the Livestock Metabolome Database ^7,43,44^ were used for the identification of metabolites. Finally, the RA of every single metabolite identified was calculated by adding up the relative concentration of peaks belonging to the same metabolite.

### Statistical analysis

Live weight change and MLB intake were averaged across the 3 days prior to each blood sampling day because this could better reflect the metabolic status of animals at that point in time ^45^. Details of LW and MLB intake data curation can be found in^11^ and^10^, respectively.

The RA of glutamine, formate, glycine, dimethyl sulfone and lactate were logarithmically transformed prior to analysis to normalise their distribution. Principal component analysis (PCA) was conducted on the RA of all identified metabolites. Only those PC with eigenvalues > 1 were selected for the final PCA and loading and score plots were drawn for the first two PC to visualise the loading of each metabolite on each PC, the correlations between metabolites, and the potential clustering of data points according to the day of blood sampling.

The association between the RA of metabolites and LWC and MLB intake at each point in time was investigated using mixed-effects linear regression models including Day and Sex as fixed effects, and LWC and MLB as covariates, and all possible interactions. Day was included as repeated measures and animal as the subject random factor. Spatial power covariance structure was used based on the lowest Bayesian Information Criterion which accounted for the distance between any two repeated measures. The model hypothesis was that the association between growth rate animals and the RA of metabolites was different at each sampling point. Differences between least-square means for the main effect of sampling day were corrected for multiple comparisons using Bonferroni test. Significant statistical differences were declared at *P* ≤ 0.05. All statistical analyses were done using SAS 9.4 (SAS Institute Inc., Cary, New Jersey, USA).

### Animal ethics

The study had animal ethics approval from The University of Sydney Animal Ethics Committee: Protocol no. 2017/1162. The study was undertaken following the Australian code for the care and use of animals for scientific purposes 8th Edition 2013.

## Data Availability

Data and algorithms used in this manuscript may be available from the corresponding author on request. Restrictions apply to the availability of these data, which were used under license for the current study, and so are not publicly available.
